# Stabilization of Electrospun Nanofiber Mats Used for Filters by 3D Printing

**DOI:** 10.3390/polym11101618

**Published:** 2019-10-06

**Authors:** Tomasz Kozior, Marah Trabelsi, Al Mamun, Lilia Sabantina, Andrea Ehrmann

**Affiliations:** 1 Faculty of Mechatronics and Mechanical Engineering, Kielce University of Technology, 25-314 Kielce, Poland; tkozior@tu.kielce.pl; 2 Faculty of Engineering and Mathematics, Bielefeld University of Applied Sciences, 33619 Bielefeld, Germany; marah.trabelsi@enis.tn (M.T.); al.mamun@fh-bielefeld.de (A.M.); lilia.sabantina@fh-bielefeld.de (L.S.); 3 Ecole Nationale d’Ingénieurs de Sfax, Sfax 3038, Tunisia

**Keywords:** nanofiber mat, electrospinning, water filter, 3D printing, FDM printing, adhesion, stabilization, carbonization

## Abstract

Electrospinning is a well-known technology used to create nanofiber mats from diverse polymers and other materials. Due to their large surface-to-volume ratio, such nanofiber mats are often applied as air or water filters. Especially the latter, however, have to be mechanically highly stable, which is challenging for common nanofiber mats. One of the approaches to overcome this problem is gluing them on top of more rigid objects, integrating them in composites, or reinforcing them using other technologies to avoid damage due to the water pressure. Here, we suggest another solution. While direct 3D printing with the fused deposition modeling (FDM) technique on macroscopic textile fabrics has been under examination by several research groups for years, here we report on direct FDM printing on nanofiber mats for the first time. We show that by choosing the proper height of the printing nozzle above the nanofiber mat, printing is possible for raw polyacrylonitrile (PAN) nanofiber mats, as well as for stabilized and even more brittle carbonized material. Under these conditions, the adhesion between both parts of the composite is high enough to prevent the nanofiber mat from being peeled off the 3D printed polymer. Abrasion tests emphasize the significantly increased mechanical properties, while contact angle examinations reveal a hydrophilicity between the original values of the electrospun and the 3D printed materials.

## 1. Introduction

Electrospinning can be used to create continuous nanofibers or nanofiber mats, typically with diameters in the range of tens to hundreds of nanometers [1,2,3]. In needle-based technology, a polymer solution or melt is pressed out of a syringe through a fine needle and stretched in a strong electric field, building a so-called Taylor cone while being drawn to a substrate [4]. Needleless technologies often apply rotating cylinders or wires, which are constantly coated with polymer melt or solution [5].

Due to their large surface space, such nanofiber mats are often used in applications that necessitate a large contact area of the material with the environment, such as catalyzers [6], medical wound dressing [7,8,9], biotechnological applications [10,11,12], or novel filter materials [13,14,15,16,17,18].

These fine nanofiber mats with typical areal weights of a few grams per square meter or even less, however, are mechanically not very stable [19], making an additional stabilization necessary in many cases. This is of special relevance in the case of electrospun water or oil filters [20]. Diverse approaches have been reported in the literature to solve this problem, such as heat pressing [14,21], ultrasonic welding of several nanofiber mats [22], coating the nanofiber mats [23], laminating the nanofiber mats [24], crosslinking of neighboring fibers [25], or embedding the nanofiber mats into textile composites [26].

Additive manufacturing technologies have many applications, both in the aerospace, automotive, and prototyping industries. At present, however, many research works focus on the study of mechanical properties and the dimensional accuracy of manufactured models [27,28,29]. Here, we report on a novel approach to combine both of the aforementioned technologies, using 3D printing on nanofiber mats to increase their mechanical stability.

Generally, 3D printing by fused deposition modeling (FDM) on macroscopic textile fabrics is under investigation in many research groups, aiming at increasing the adhesion between both parts of the composite by mechanical, thermal, or chemical methods [30,31,32,33,34,35,36,37,38]. Direct 3D printing on electrospun nanofiber mats prepared with different equipment, however, has, to the best of our knowledge, not yet been reported in the scientific literature. Reports of combinations of both technologies by electrospinning on 3D printed objects [39,40,41], on objects for which 3D printed negative forms were created [42], or by creating 3D printing inks from electrospun nanofibers [43], have been recently published. Only one report on a special self-built machine, created to produce alternatingly 3D-printed and electrospun layers by a needle-based method, can be found in the scientific literature [44]. Our results reported here go without such specialized equipment, but evaluate 3D printing by a common FDM printer on needleless electrospun nanofiber mats.

## 2. Materials and Methods

For electrospinning, the needleless electrospinning machine “Nanospider Lab” (Elmarco Ltd., Liberec, Czech Republic) was used by applying the following spinning parameters: high voltage 50–80 kV, nozzle diameter 0.8–0.9 mm, carriage speed 100 mm/s, bottom electrode/substrate distance 240 mm, ground electrode/substrate distance 50 mm, temperature in the chamber 22–23 °C, and relative humidity in the chamber 32–33%. As substrates, either a polypropylene nonwoven or an aluminum foil was used. On the latter, PAN nanofibers mats adhere strongly [45], allowing one to use the aluminum foil as a relatively rigid substrate during 3D printing. Spinning was carried out for 10 min (on aluminum), 20 min (in case of 70 kV), or 30 min (in case of 50 kV) to create sufficiently thick nanofiber mats.

The spinning solution was prepared from 16% polyacrylonitrile (PAN) dissolved in dimethyl sulphoxide (DMSO, min. 99.9%, purchased from S3 Chemicals, Bad Oeynhausen, Germany). PAN was used for this proof-of-concept, since it can be electrospun from the low-toxic DMSO [46] and has been investigated in detail during stabilization and carbonization before [47].

Some nanofiber mats were stabilized in a muffle furnace B150 (Nabertherm, Lilienthal, Germany), at a typical stabilization temperature of 280 °C approached with a heating rate of 1 K/min, followed by isothermal treatment at this temperature for 1 h. Carbonization was performed in a furnace (Carbolite Gero, Neuhausen, Germany) at a temperature of 500 °C, reached with a heating rate of 10 K/min in a nitrogen flow of 150 mL/min (STP) and isothermal treatment at this temperature for 1 h.

**Figure 1:**
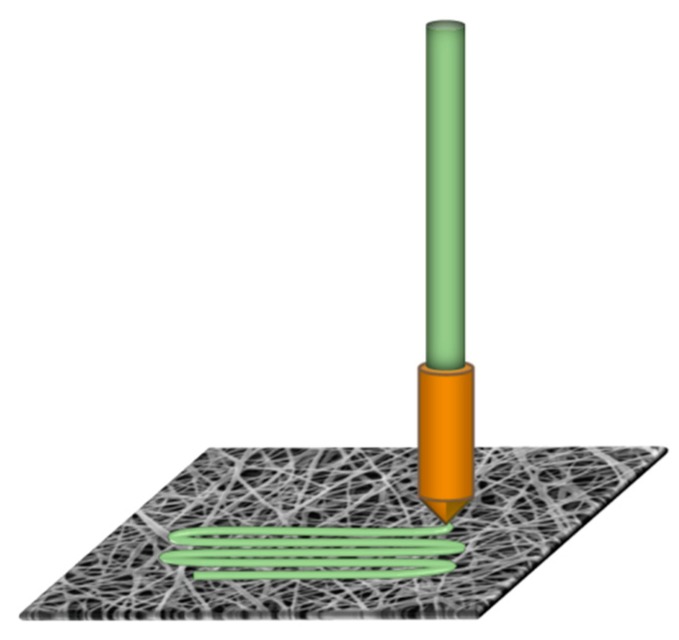
3D printing with fused deposition modeling (FDM) technology on a polyacrylonitrile (PAN) nanofiber mat. The printing polymer PLA (here green) is delivered as a filament into the nozzle in a molten state and placed on the nanofiber mat.

On these nanofiber mats, 3D printing was performed using the FDM printer Orcabot XXL (Prodim, The Netherlands) with a nozzle diameter of 0.4 mm, using a layer thickness of 0.2 mm, a nozzle temperature of 190 °C to 210 °C, and different printing bed temperatures between room temperature and 80 °C. The polymer material was poly(lactic acid) (PLA) (purchased from Filamentworld, Neu-Ulm, Germany). A sketch of this process is presented in Figure 1.

For the optical and chemical evaluation of the composites, we used a digital microscope VHX-600D (Keyence, Neu-Isenburg, Germany), a confocal laser scanning microscope (CLSM) VK-8710 (Keyence), and an Excalibur 3100 (Varian, Inc., USA) FTIR spectrometer.

Contact angles were investigated by placing drops of distilled water with a volume of 15 µL onto the samples under examination, taking microscopic images with the aforementioned digital microscope and fitting the angles between the drop contour and the baseline between object and drop.

The abrasion resistance of the nanofiber mats on the 3D printed polymer surface was investigated by a Martindale abrasion tester, working according to ISO 12947, and evaluating the damage on the surface by eye, as defined in the standard, as well as with the aforementioned digital microscope.

## 3. Results and Discussion

**Figure 2:**
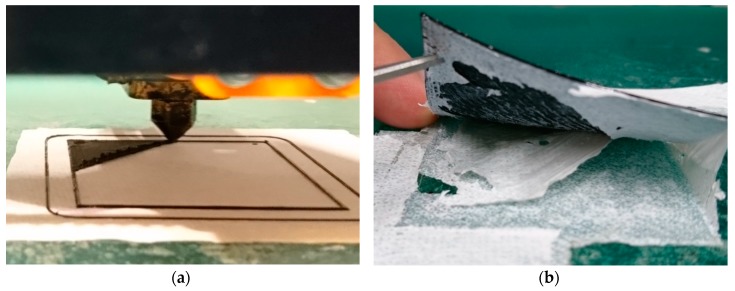
3D printing squares of dimensions 40 mm × 40 mm on nanofiber mats: (**a**) rough surface for a too large distance between nanofiber mat and printing nozzle and (**b**) composite strongly sticking on the double-sided adhesive tape used for gluing the nanofiber mat over its entire area.

Generally, 3D printing on nanofiber mats was found to require that the distance between printing nozzle and nanofiber mat was controlled exactly. On the one hand, the nanofiber mat breaks at once if the distance is too small or the nozzle even touches the mat, which is opposite to 3D printing on woven, warp knitted, or weft knitted fabrics, where it can be advantageous in terms of adhesion for pressing the filament into the textile by printing “below” the textile surface [31]. On the other hand, if the distance is too large, the contact between both materials is lost, resulting in a very uneven surface (Figure 2a). It must be mentioned that gluing the nanofiber mat onto the printing bed over its entire area results in severe problems to detach the composite from the printing bed afterwards (Figure 2b) and thus cannot be recommended.

In a first test series, nanofiber mats were glued along their borders onto the printing bed, and the printing bed temperature was modified from room temperature to 40 °C, 60 °C, and 80 °C. While a temperature of 60 °C or higher strongly supports the adhesion of a 3D printed PLA layer on the printing bed, here we found that printing at room temperature or at 40 °C showed similar results to using a printing bed temperature of 60 °C, while a temperature of 80 °C resulted in severe problems, prohibiting us from finding a suitable nozzle-nanofiber mat distance in which none of the aforementioned problems occurred. Since heating the printing bed did not show any advantage, the results depicted here were gained with the printing bed at room temperature.

Similarly, different nozzle temperatures between 190 °C and 210 °C were tested, which are well-suited to print PLA. Former experiments revealed that higher temperatures allowed for 3D printing on textile fabrics in a larger distance to reach the same adhesion [48]. Here, however, the possible distance range that was sufficient to create reliable adhesion without breaking the nanofiber mat could not be extended in this way.

Detailed observation of the printing process if the nozzle is slightly too high suggests that the problem of missing adhesion for too large distances between nanofiber mat and printing nozzle may be based on electrostatic repulsion between the 3D printing polymer and the nanofiber mat. In a previous experiment, the electrostatic charging could be significantly reduced by soaking the nanofiber mat into water with a surfactant that was typically negatively charged [49]. Thus, 3D printing was also tested on a nanofiber mat that was soaked in soap water and dried in the air.

**Figure 3:**
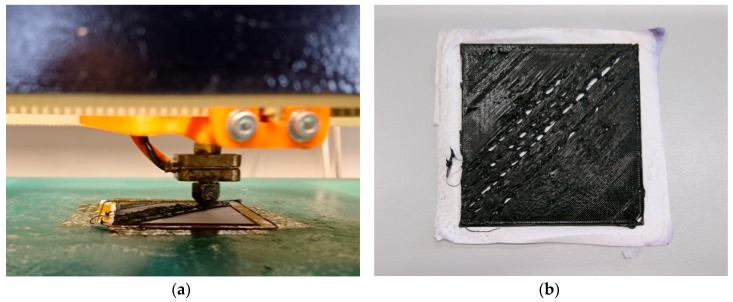
3D printing squares of dimensions 40 mm × 40 mm on nanofiber mats previously dipped in soap water: (**a**) rough surface for a too large distance between nanofiber mat and printing nozzle; (**b**) 3D printed layer with uneven surface and even several not closed areas near the diagonal, i.e., along the longest lines, due to a too large distance between nozzle and nanofiber mat.

In this experiment, however, we found that controlling the distance between nozzle and nanofiber mat was not easier, and the danger of reduced contact between 3D printed polymer and nanofiber mat could not be reduced in this way. Figure 3 depicts an example of a slightly too high nozzle, resulting in a rough surface or even open areas in the 3D printed surface for the longest lines near the diagonal of the sample.

On the other hand, soaking and drying the sample in pure water or soap water before printing on it supports relaxation [49]. In this way, fixing it in a slightly stretched position was simpler than in case of not watered samples, in order to ensure that no elongation of the specimen was possible due to thermal and mechanical impact during printing. Apparently, in spite of the unreached original goal, this pre-treatment is clearly advantageous and should thus always be performed before 3D printing on nanofiber mats.

**Figure 4:**
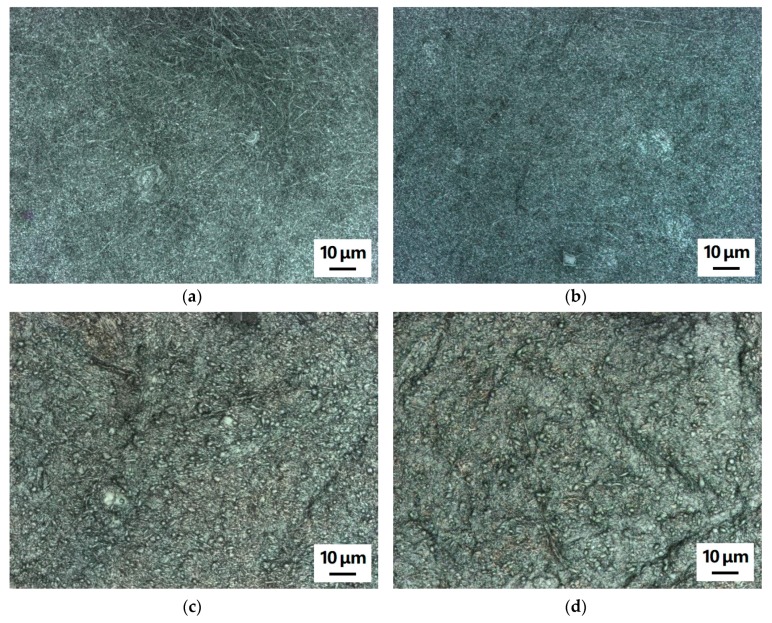
Confocal laser scanning microscope (CLSM) images of polyacrylonitrile (PAN) nanofiber mats: (**a**) pure PAN (electrospun with 80 kV), (**b**) pure PAN (electrospun with 80 kV) after printing on the other side of the nanofiber mat, (**c**) carbonized PAN (electrospun with 50 kV), and (**d**) carbonized PAN (electrospun with 50 kV) after printing on the other side of the nanofiber mat.

Next, the question arises whether 3D printing with a nozzle temperature of approx. 200 °C may influence the morphology of the nanofiber mat. This is important for the possible application of the nanofiber composites as filter materials. Figure 4 depicts examples of CLSM images, taken on pure nanofiber mats and on nanofiber mats after 3D printing on their backsides to enable comparison. Neither the pure PAN nanofiber mats (Figure 4a,b) or the carbonized ones (Figure 4c,d) show any difference in the morphology. In addition, no color change is visible for the pure PAN nanofiber mat, which might occur for temperature treatment at 180 °C or higher due to the stabilization of the nanofibers [50]. Here, however, either the duration of the temperature treatment is not long enough or the polymer melt touching the nanofiber mat has a temperature below 180 °C so that no stabilization process can start.

**Figure 5:**
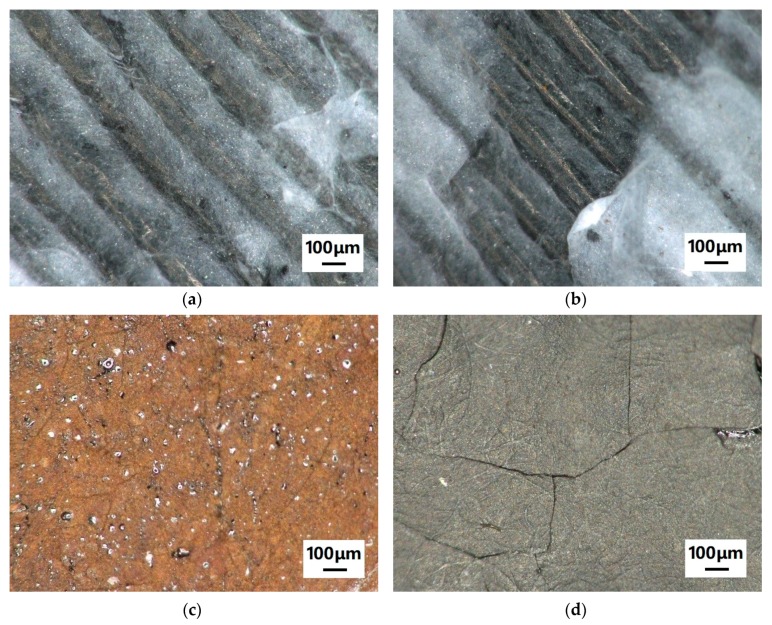
Optical microscopic images of PAN nanofiber mats after 3D printing and being peeled off the printed polymer: (**a**) PAN on aluminum (electrospun with 80 kV)—average situation; (**b**) PAN on aluminum (electrospun with 80 kV)—thinnest nanofiber “coating” found on all samples; (**c**) stabilized PAN (electrospun with 80 kV); and (**d**) carbonized PAN (electrospun with 50 kV).

After 3D printing on the nanofiber mats, the composites were detached from the printing bed, and, starting at the edges, the nanofiber mats were carefully peeled from the 3D printed polymer to investigate whether the adhesion between 3D printing polymer and nanofiber mat was stronger or smaller than the adhesion of the layers inside the nanofiber mat. Figure 5 depicts exemplary images of the residues of the nanofiber mats, showing areas with thicker or thinner nanofiber layers left after peeling them off. In the case of the pure PAN nanofiber mats (Figure 5a,b), it is clearly visible that the nanofiber mats were separated inside the fabrics, while even in the areas with the thinnest nanofiber coatings, no positions were found where the nanofiber mats were fully separated from the 3D printing polymer.

This shows clearly that the adhesion between 3D printing polymer and nanofiber mat is uncritical. For better bonding of the fibers inside the mat, either stabilization at higher heating rates than used here [45,50] or crosslinking of the fiber connection points by exposing the nanofiber mat to a solvent vapor [25] can be applied.

The impact of stabilization—even for the small heating rate used here, which does not significantly modify the nanofiber morphology and does not lead to visible conglutinations at the crossing points of neighboring nanofibers—is visible in Figure 5c,d. Thick layers of the stabilized and the carbonized nanofiber mats remain after trying to peel them off, which are, on the other hand, prone to showing micro-cracks, as visible in Figure 5. This suggests either using a chemical method to bond the nanofibers inside the mats better [25] or to fix the whole nanofiber mats on the 3D printed polymer by printing an open grid on the nanofiber mats after the first printing step. Due to the strong relaxation of nanofiber mats during evaporation of residual solvent [49], the opposite procedure of electrospinning on 3D printed layers does not seem to be suitable, but can be expected to result in breaking of the nanofiber mats, which may also happen during electrospinning on the commonly used polypropylene nonwoven.

**Figure 6:**
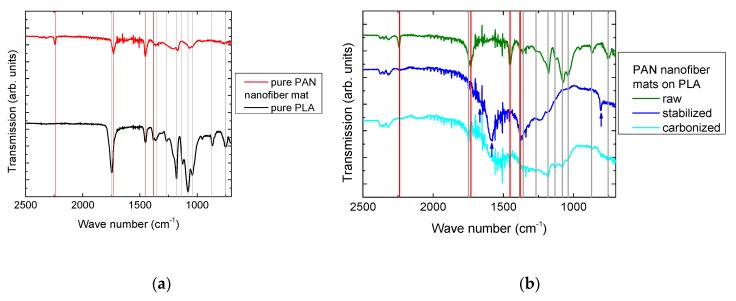
FTIR spectra of (**a**) pure PAN and pure poly(lactic acid) (PLA) for comparison, with some prominent peaks marked and (**b**) PAN nanofiber mats in raw, stabilized, and carbonized state on PLA.

Next, Figure 6b shows FTIR measurements of PAN nanofiber mats on 3D printed PLA. Figure 6a gives an overview of typical peaks of PLA [51,52,53,54] (grey lines) and PAN [55] (red lines), respectively.

In Figure 6b, the curve measured for the raw PAN nanofiber mat clearly shows peaks of both PLA (grey lines) and PAN (red lines), indicating the composite character of the surface under examination. For the stabilized samples, new peaks can be expected at 1582 cm^−1^, 1660 cm^−1^, and approx. 800 cm^−1^ [55,56,57]. Here, these peaks are clearly visible (blue arrows), while no PLA peaks can be recognized due to the thick nanofiber mat on top of the PLA layer. Finally, for the carbonized layer, the characteristic peaks of the stabilized material are usually nearly completely vanished due to the high absorbance of carbon, leaving only very few functional groups [58]. Again, the peaks resulting from PLA are nearly vanished due to the almost complete carbon nanofiber mat on top of the PLA material.

**Figure 7:**
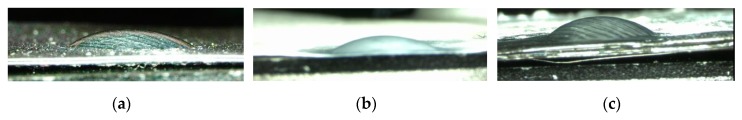
Contact angles, investigated by dropping 15 µL of distilled water on (**a**) an FDM-printed PLA layer, (**b**) an electrospun PAN nanofiber mat, and (**c**) a PAN/PLA composite prepared as described in this article.

To investigate the hydrophobic properties of the original PAN nanofiber mat, the PLA 3D printing material and the composite contact angle measurements are performed (Figure 7). Since all materials are strongly hydrophilic, the most crucial point is the time between setting the drop on the object under examination and taking the photograph. Contact angles measured approx. 1 s after setting the drop are (34 ± 3)° for PLA, (31 ± 3)° for PAN, and (32 ± 4)° for the PAN/PLA composite surface.

While the contact angles thus do not show a significant difference, it should be mentioned that the drop vanishes slightly faster on the pure PAN nanofiber mat, as compared to the composite, and stays constant for the closed PLA surface. It is observed that spreading of the water inside the nanofiber mat was unidirectional for the pure PAN nanofiber mat, while it was guided along the printing lines in case of the composite. This underlines that care must be taken to position such a composite filter in the optimal orientation with respect to the water flow.

**Figure 8:**
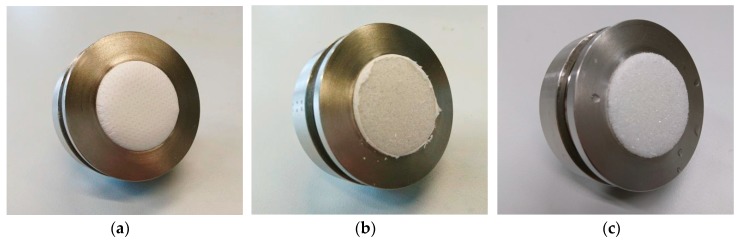
Electrospun nanofiber mat in Martindale abrasion test holder, (**a**) before the test, (**b**) after 10 cycles in dry state, and (**c**) after one cycle in wet state.

Finally, the mechanical properties of the composites are investigated by a Martindale abrasion test (Figure 8a). This test is usually performed with macroscopic textiles; thus, it destroyed the nanofiber mat in the dry state between 5 and 10 Martindale cycles (Figure 8b) and in the wet state after the first cycle (Figure 8c).

**Figure 9:**
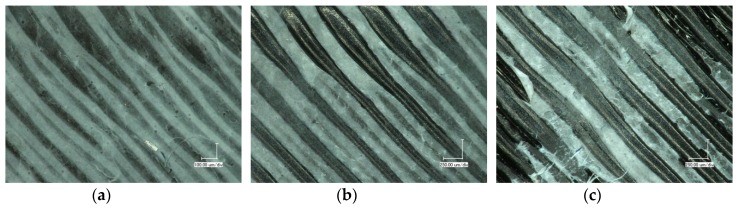
Composite surface after Martindale abrasion tests, (**a**) before the test, (**b**) after 10 cycles in dry state, and (**c**) after 50 cycles in dry state.

This changed dramatically for the composites surfaces. Figure 9 depicts exemplary images of these surfaces before the tests, after 10, and after 50 Martindale cycles. While after 10 cycles, first protruding areas are abraded, after 50 cycles parts of the nanofiber mats on narrower areas that were not properly fixed there are torn apart; thus, the test was stopped. It should be mentioned that this behavior was quantitatively identical for tests on wet composites. In addition, several areas were still fully coated with the nanofiber mat after 500 Martindale cycles.

These experiments underline that fixing the nanofiber mats by 3D printing on them is generally possible. Nevertheless, further investigations are necessary to optimize the adhesion between both materials over the whole contact area without increasing the danger of touching and thus destroying the nanofiber mat with the 3D printing nozzle.

## 4. Conclusions

To conclude, we have successfully tested the possibility to prepare composites by 3D printing on raw, stabilized, or carbonized PAN nanofiber mats, thus mechanically stabilizing the nanofiber mats. Optical and chemical examinations revealed that the nanofiber mats were not measurably modified by the 3D printing process. Contact angle examinations did not show significant differences in hydrophilicity, comparing the pure nanofiber mat and the composite surface. Martindale abrasion tests underlined the significantly increased abrasion resistance of the composite. 

While in this first proof-of-principle, a full layer was 3D printed on the nanofiber mats, as the composites could be used in filter applications with the liquid flow parallel to them, future experiments have to be conducted to investigate the possibility to print open mesh-like structures on nanofiber mats to also enable utilization in filters through which the liquid flows.
